# Temperature-dependent differences in mouse gut motility are mediated by stress

**DOI:** 10.1038/s41684-024-01376-5

**Published:** 2024-05-28

**Authors:** Alvin Han, Courtney Hudson-Paz, Beatriz G. Robinson, Laren Becker, Amanda Jacobson, Julia A. Kaltschmidt, Jennifer L. Garrison, Ami S. Bhatt, Denise M. Monack

**Affiliations:** 1 Department of Microbiology and Immunology, Stanford University, Stanford, CA USA; 2 Buck Institute for Research on Aging, Novato, CA USA; 3 Neurosciences IDP Graduate Program, Stanford University School of Medicine, Stanford, CA USA; 4 Department of Medicine (Gastroenterology and Hepatology), Stanford University, Stanford, CA USA; 5 Genentech Inc., Research and Early Development, Immunology Discovery, South San Francisco, CA USA; 6 Department of Neurosurgery, Stanford University School of Medicine, Stanford, CA USA; 7 Global Consortium for Reproductive Longevity & Equality, Novato, CA USA; 8 Department of Medicine (Hematology, Blood and Marrow Transplantation), Stanford University, Stanford, CA USA; 9 Department of Genetics, Stanford University School of Medicine, Stanford, CA USA

**Keywords:** Microbiota, Gastrointestinal hormones, Gastrointestinal models, Gastrointestinal system, Mouse

## Abstract

Researchers have advocated elevating mouse housing temperatures from the conventional ~22 °C to the mouse thermoneutral point of 30 °C to enhance translational research. However, the impact of environmental temperature on mouse gastrointestinal physiology remains largely unexplored. Here we show that mice raised at 22 °C exhibit whole gut transit speed nearly twice as fast as those raised at 30 °C, primarily driven by a threefold increase in colon transit speed. Furthermore, gut microbiota composition differs between the two temperatures but does not dictate temperature-dependent differences in gut motility. Notably, increased stress signals from the hypothalamic–pituitary–adrenal axis at 22 °C have a pivotal role in mediating temperature-dependent differences in gut motility. Pharmacological and genetic depletion of the stress hormone corticotropin-releasing hormone slows gut motility in stressed 22 °C mice but has no comparable effect in relatively unstressed 30 °C mice. In conclusion, our findings highlight that colder mouse facility temperatures significantly increase gut motility through hormonal stress pathways.

## Abstract (web summary)

The study reveals that raising mice at 22 °C boosts gut transit speed by two times compared to 30 °C, primarily due to stress signals from the hypothalamic–pituitary–adrenal axis and an increase of corticotropin-releasing hormone.

## Main

Mice (*Mus musculus*) are the most commonly used models for human disease and biology. Despite the myriad of important physiological and disease-related discoveries that have been made using mouse model systems, model organism research has drawbacks. Increasingly, scientists have raised concerns about the failures of mouse research to translate into clinical outcomes and to reproduce across and even within institutions^1^. These issues have become prevalent enough to prompt the National Institutes of Health to recommend funding research that investigates the root causes of these failures. In the 2021 working group report, they highlight the need to understand how environmental factors contribute to difficulties in the translation of mouse research findings to humans^1^. One factor that may contribute to the failure of translation in mouse research that is gaining increased attention is the choice of housing temperature in animal facilities.

So far, there has not been a sufficiently compelling rationale for an ideal mouse housing temperature for the purposes of translational research. The use of ~22 °C as the standard housing temperature is not evidence based, and it is largely believed to be based on comfortable working temperatures for humans^2^. In light of this, a growing body of evidence has accumulated and now suggests that 22 °C housing induces chronic cold stress in mice^3^. This cold stress manifests as increased metabolism, heart rate and blood pressure and is associated with altered immune function and increased tumor growth^2,4–10^. Cold stress is alleviated at thermoneutrality, a range of housing temperatures at which an animal’s metabolic rate is minimal and constant^10^. For mice, this temperature is between 29 °C and 33 °C, with most studies of thermoneutral mouse biology adopting 30 °C as the thermostat set point^10^. Humans spend most of their time at thermoneutrality due to better thermoregulatory mechanisms such as larger body mass, shelters or clothing^11,12^. Based on this, thermoneutral mice have been proposed as superior translational models compared to mice housed at 22 °C (refs. ^2,3,13^). Indeed, many studies show that 30 °C mice better model humans than 22 °C mice in a variety of contexts^3,14–19^. Together, these data strongly suggest that improving the translatability of mouse model research outcomes could be achieved by changing the housing temperature from ~22 °C to 30 °C. While initial experiments, such as those outlined above, support adjusting the housing temperatures of laboratory mice to a higher temperature, our understanding of the impact of ambient temperature on many aspects of mouse physiology is very limited. A more detailed catalog of how phenotypes change in mice at 30 °C will be critical for better assessing the suitability of thermoneutral mice as translational models.

Notably, several basic physiological functions of the gut, such as motility, remain uncharacterized in thermoneutral mice. To our knowledge, the only studies on the effects of ambient temperature on gut motility have shown contrasting results in nonmodel organisms; one study found that dogs had faster gastric emptying in cold temperatures, while another found that young chickens displayed slower gastrointestinal motility in cold temperatures except in the esophagus and crop^20,21^. Temperature has never been linked to gut motility changes in mice, but previous studies suggest that a potential link between environmental temperature and gut motility could be the hypothalamic–pituitary–adrenal (HPA) axis stress response. Studies have shown that cold exposure causes the production of corticosterone, the end hormone product of the HPA axis stress response, in rodents^14,22^. Additionally, administration of restraint stress or corticotropin-releasing hormone (CRH), the initial HPA axis stress response hormone, induced changes in rat gut motility^23^, and administration of psychological stress to mice produced elevated corticosterone and gut dysmotility^24^. Therefore, stress impacts gut motility, and temperature is linked to changes in stress levels, but previous studies have not directly linked the effects of different temperatures on gut motility^14,22^. Thus, resolving the links between temperature, stress and gut motility would broadly provide useful insights into the relationship between environmental variables and organismal biology while also informing the ongoing debate about ‘ideal’ mouse facility temperatures.

In this Article, we sought to determine if and how cold stress affects the gut motility of mice. First, we showed that the whole gut transit time (WGTT) of 22 °C mice is substantially faster than that of 30 °C mice, which is primarily driven by faster colonic motility at 22 °C. Second, we find that while the gut microbiota composition of 22 °C and 30 °C mice differs at a species level, it does not drive temperature-dependent differences in gut motility. We further demonstrate that the molecular link between ambient temperature and gut motility involves the HPA axis stress response. More specifically, mice at 22 °C have elevated stress hormone levels compared to 30 °C mice. We also find that pharmacologic intervention and genetic removal of the stress neuropeptide CRH are sufficient to ablate these differences. Taken together, our findings illustrate a mechanism by which environmental temperatures, and specifically thermoneutrality, alter the gut motility of mice. Ultimately, these findings further illuminate environmental effects on organismal biology and inform efforts to eliminate translational and reproducibility errors in mouse research.

## Results

### Gut motility differs on the basis of environmental temperature

**Fig. 1:**
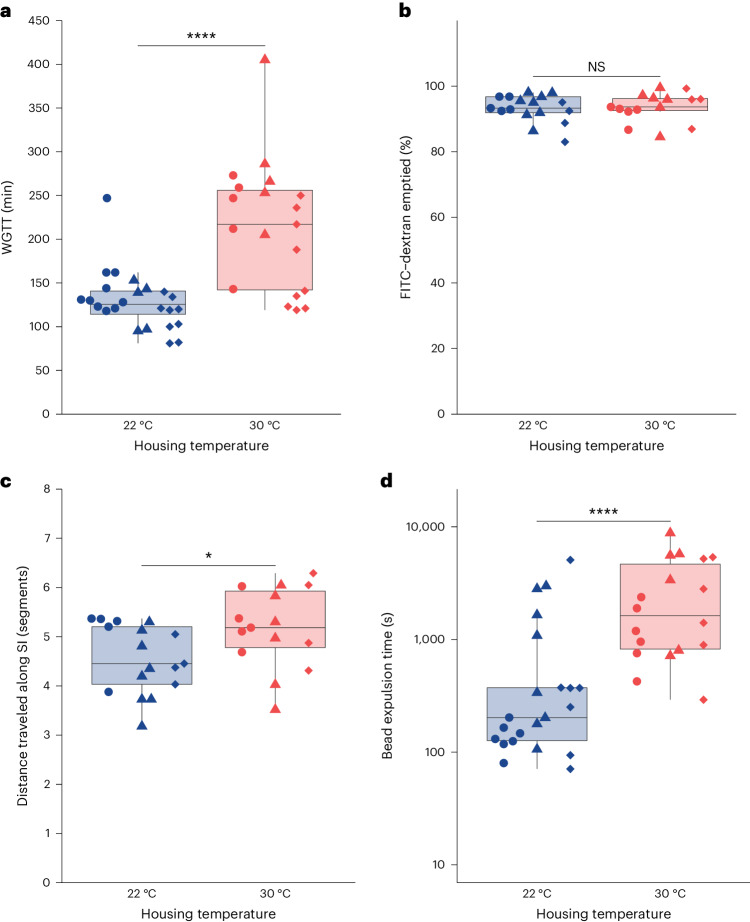
Gut motility of mice differs depending on ambient temperature. Gut motility measurements were performed on 22 °C C57BL/6 mice (blue) or 30 °C mice (red). Each point represents a measurement from an individual mouse. Shapes (circle, diamond and triangle) indicate the independent experiment in which a measurement was taken. Boxes indicate the upper and lower quartiles, midline represents the median and whiskers indicate nonoutlier minima and maxima. Significance test results indicated by: NS *P* > 0.05, *P < 0.05, **P < 0.01, ***P < 0.001, ****P < 0.0001. Extended statistics can be found in Supplementary Table 1. **a**, WGTT of 22 °C mice (blue) or 30 °C mice (red) was determined by the time difference between oral gavage and defecation of carmine red dye (*N*_22°C_ = 24 (13 F, 11 M), *N*_30°C_ = 19 (10 F, 9 M); Wilcoxon test, *W* = 388, *P* = 9.54 × 10^−5^). **b**, Gastric emptying time was determined by the percentage of a FITC–dextran solution emptied in 30 min from stomachs of 22 °C mice (blue) or 30 °C mice (red) (*N*_22°C_ = 17 (7 F, 10 M), *N*_30°C_ = 15 (7 F, 8 M); Wilcoxon test, *W* = 115, *P* = 0.655). **c**, SI transit was determined by tracking the distance traversed by FITC–dextran 30 min after it was administered via oral gavage to 22 °C mice (blue) or 30 °C mice (red). SI motility is expressed as the number of equidistant segments of the SI traversed by the geometric center of the FITC–dextran bolus (*N*_22°C_ = 17 (7 F, 10 M), *N*_30°C_ = 15 (7 F, 8 M); *t*-test, *t* = −2.30, d.f. 27.8, *P* = 0.0293). **d**, Colon transit was determined by measuring the time it took 22 °C mice (blue) or 30 °C mice (red) to expel a 3 mm glass bead gently inserted 2 cm into the distal colon (*N*_22°C_ = 22 (11 F, 11 M), *N*_30°C_ = 18 (7 F, 11 M); Wilcoxon test, *W* = 57, *P* = 5.34 × 10^−5^). F, female; M, male. Source data

We first sought to investigate whether the gut motility of C57BL/6 mice raised at 22 °C and 30 °C, referred to hereafter as 22 °C mice and 30 °C mice, respectively, differed. We elected to study this inbred strain due to its ubiquity and its status as the most commonly used background for maintaining mutant lines^25^. To evaluate differences in gut motility, we first performed a standard WGTT assay, in which we measured the amount of time between oral gavage of carmine red dye and passage of the first red fecal pellet. Remarkably, we found that 22 °C mice had a WGTT roughly twice as fast as that of 30 °C mice (Fig. 1a). When we stratified these results by sex, we found no sex-dependent effect (Supplementary Fig. 1a). We also performed this assay in 129×1/SvJ mice and found that 22 °C mice had a WGTT roughly twice as fast as 30 °C mice (Supplementary Fig. 1b), suggesting that this is not a strain-specific effect.

Next, we determined whether the whole gut transit phenotype was attributable to a particular compartment of the gut by performing assays that specifically measure transit times in the stomach, small intestine (SI) and colon. To measure gastric emptying speed, we orally gavaged mice with fluorescein isothiocyanate (FITC)–dextran 70 kDa and determined what percentage was cleared from the stomach after 30 minutes. We found no difference in the percentage of FITC–dextran cleared from the stomach in 22 °C and 30 °C mice (Fig. 1b), indicating that gastric emptying is not affected by housing temperature. Next, we measured small intestinal transit using two methods. In the initial approach, we measured the distance along the SI that the geometric center of the fluorescence distribution of the FITC–dextran 70 kDa traveled 30 min after oral gavage (Fig. 1c). As a complementary approach, we measured the distance traveled by the leading edge of a bolus of activated charcoal 30 min after oral gavage (Supplementary Fig. 2). We found that 22 °C mice had slower small intestinal motility than 30 °C mice by both measures (Fig. 1c and Supplementary Fig. 2). Finally, we used a standard colon bead expulsion assay to assess transit time differences in the colon by measuring the time it took a mouse to excrete a glass bead inserted 2 cm into the distal colon. We found that distal colon transit time was roughly three times as fast at 22 °C as compared to 30 °C (Fig. 1d). Thus, we demonstrate that the temperature that mice are raised at impacts gut motility and that there are compartment-specific differences in transit time. Collectively, our data demonstrate that, although 22 °C mice had slightly slower small intestinal transit, their much faster colon transit drove the overall faster WGTT at 22 °C compared to 30 °C.

### The gut microbiota differs in 22 °C and 30 °C mice but does not drive differences in temperature-dependent gut motility

**Fig. 2:**
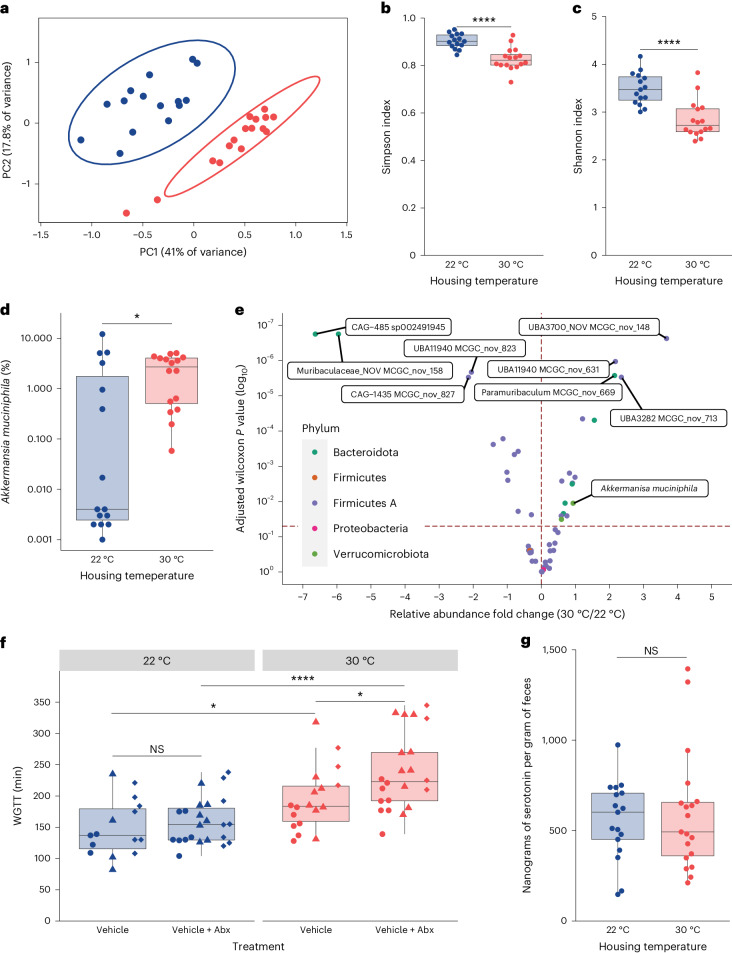
Mouse gut microbiome composition differs depending on ambient temperature. Shotgun-metagenomic sequencing was performed on DNA extracted from fecal pellets of 22 °C mice or 30 °C C57BL/6 mice (*N*_22°C_ = 15 (6 F, 9 M), *N*_30°C_ = 16 (9 F, 7 M); **a**–**e**). Reads were classified and subsequently analyzed for differences in beta diversity, alpha diversity and enrichment of bacterial species. Each point (except in **a** and **e**) represents a measurement from an individual mouse. Shapes (circles, diamonds and triangles) indicate the independent experiment in which a measurement was taken. Boxes indicate the upper and lower quartiles, midline represents the median and whiskers indicate nonoutlier minima and maxima. Significance test results indicated by NS *P* > 0.05, *P < 0.05, **P < 0.01, ***P < 0.001, ****P < 0.0001. Extended statistics can be found in Supplementary Table 1. **a**, Principal coordinates (PC) analysis of fecal gut microbiomes of 22 °C mice (blue) or 30 °C mice (red) using Bray–Curtis dissimilarity as a distance metric. The points represent fecal microbiomes of individual mice. A PERMANOVA was performed using housing temperature as the grouping variable (*F* = 13.710, *P* = 0.001). **b**, Alpha diversity of fecal microbiomes collected from 22 °C mice (blue) or 30 °C mice (red) was measured by Simpson index (*t*-test, *t* = 5.24, d.f. 26.0, *P* = 1.80 × 10^−5^). **c**, Alpha diversity of fecal microbiomes collected from 22 °C mice (blue) or 30 °C mice (red) was measured by Shannon index (*t*-test, *t* = 4.94, d.f. 28.7, *P* = 3.11 × 10^−5^). **d**, The relative abundance of *A. muciniphila* reads in fecal microbiomes of 22 °C mice (blue) or 30 °C mice (red) (Wilcoxon test, *W* = 66, *P* = 0.0331). **e**, Volcano plot of differentially enriched species present in at least 30% of samples at 0.1% or more relative abundance identified using Benjamini–Hochberg-corrected *P* values from ALDEx2. The points are colored by phylum. Organisms enriched in 30 °C mice have a positive fold change along the *x* axis. The dotted line on the *x* axis denotes no difference in relative abundance, and the dotted line on the *y* axis denotes *P*_adj_ = 0.05. **f**, WGTT of 22 °C mice (blue) or 30 °C mice (red) treated for 2 weeks with either vehicle or vehicle plus antibiotics (Abx) as determined by time difference between gavage and defecation of carmine red dye (*N*_22°C,Veh_ = 15 (9 F, 6 M), *N*_22°C,Veh+Abx_ = 23 (16 F, 7 M), *N*_30°C,Veh_ = 18 (6 F, 12 M), *N*_30°C,Veh+Abx_ = 22 (15 F, 7 M); *t*-tests; vehicle 22 °C versus 30 °C: *t* = −2.70, d.f. 30.8, *P* = 0.0111; vehicle + Abx 30 °C versus 22 °C: *t* = −5.19, d.f. 34.1, *P* = 9.66 × 10^−6^; 22 °C vehicle versus vehicle + Abx, *t* = −0.713, d.f. 25.6, *P* = 0.482; 30 °C vehicle versus vehicle + Abx: *t* = −2.45, d.f. 38.0, *P* = 0.0189). **g**, Fecal concentration of serotonin normalized to wet weight of feces 22 °C mice (blue) or 30 °C mice (red) as determined by ELISA (*N*_22°C_ = 17 (9 F, 8 M), *N*_30°C_ = 19 (11 F, 8 M); *t*-test, *t* = −0.356, d.f. 31.3, *P* = 0.725). Source data

We next sought to identify a mechanism for temperature-dependent differences in gut motility. Previous studies have shown that the gut microbiota can modulate gut motility^26–28^ and that mouse housing temperature can impact gut microbiota composition^14,29,30^. Thus, we first examined whether gut microbiota composition differed between 22 °C mice and 30 °C mice. We performed shotgun-metagenomic sequencing on fecal samples collected from mice and classified the reads against the Mouse Gastrointestinal Bacteria Catalogue^31^. Principal coordinate analysis using Bray–Curtis distances and permutational multivariate analysis of variance (PERMANOVA) analysis using temperature as the grouping variable revealed that the gut microbiota compositions differ significantly by housing temperature (*P* = 0.001; Fig. 2a). In addition, 22 °C mice have higher alpha diversity compared to 30 °C mice, as indicated by both Simpson and Shannon indices (Fig. 2b,c). We also performed enrichment analysis of bacterial species with ALDEx2 and identified 29 differentially enriched bacterial species (Fig. 2e). Interestingly, we noticed a reduced relative abundance of *Akkermansia muciniphila* in 22 °C mice (Fig. 2d,e), which has been associated with faster gut motility in humans^32–34^. Thus, the gut microbiota composition of 22 °C mice differs from that of 30 °C mice and shows signatures of faster gut motility.

We next tested whether the gut microbiota contributed to the gut motility phenotypes we observed. To do so, we treated mice raised at 22 °C and 30 °C with either a vehicle solution or an antibiotic cocktail in drinking water for 2 weeks to ablate the gut microbiota (Supplementary Fig. 3), then performed a WGTT assay. We found both vehicle-only and vehicle plus antibiotic-treated 22 °C mice had faster gut motility compared to vehicle-only and vehicle plus antibiotic-treated 30 °C mice, respectively (Fig. 2f). Interestingly, we observed a small but statistically significant slowing of gut motility in antibiotic-treated versus vehicle-treated 30 °C mice but not in 22 °C mice. Consistent with these findings, there was no difference in the concentration of serotonin, a microbiome-regulated hormone that modulates gut motility^28^, in the feces from 22 °C and 30 °C mice (Fig. 2g). Taken together, our results suggest that the gut microbiota is not primarily responsible for the observed temperature-dependent differences in gut motility.

### Temperature-dependent gut motility differences are not driven by food intake

Consuming food can activate reflexes that influence gastrointestinal activities such as motility^35^. Various factors can influence the amount of food consumed by mice. For example, a previous study showed that 8-week-old singly housed mice consume different amounts of food at 22 °C compared to 30 °C after a 3-week acclimation period^36^. However, Maher et al. showed that group-housed mice raised at 20 °C and 30 °C do not consume different amounts of food^37^. Thus, to explore the possibility that increased food consumption at 22 °C could drive increased whole gut motility in our system, in which mice are group-housed rather than singly housed, we measured the average food intake of 22 °C and 30 °C mice. We found no statistically significant difference in food intake when comparing mice raised at 22 °C and 30 °C, regardless of sex (Supplementary Fig. 4). Food intake also dictates weight gain, so we measured the weight of 22 °C and 30 °C mice, finding only a slight elevation in the body mass of female 30 °C mice and similar weight gain trajectories over time (Supplementary Fig. 5). Given the dramatic difference we observe in WGTT, we concluded that neither differential intake of food nor gross differences in body mass was likely to drive temperature-dependent differences in gut motility.

### Acclimation to a new temperature is sufficient to change gut motility

**Fig. 3:**
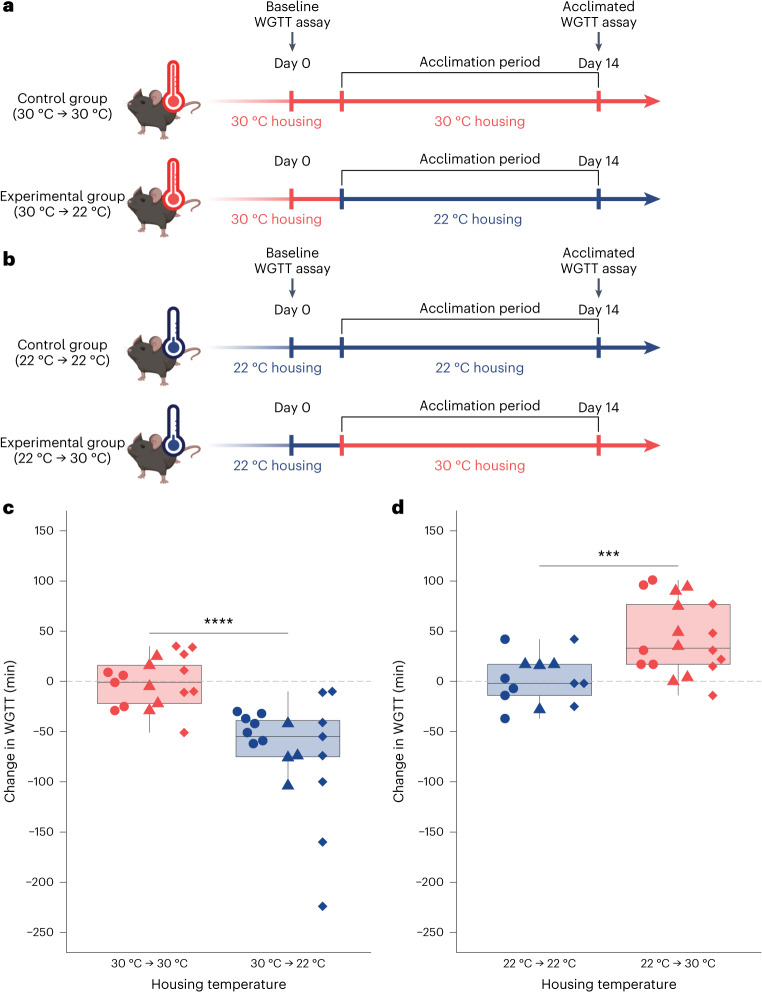
Gut transit time is altered by acclimation to a different housing temperature. Difference in WGTT before and after an acclimation period of 2 weeks to the same temperature, or a shift to a different temperature, for individual C57BL/6 mice determined by carmine red dye assay. Each point represents a measurement from an individual mouse. Shapes (circles, diamonds and triangles) indicate the independent experiment in which a measurement was taken. Boxes indicate the upper and lower quartiles, midline represents the median and whiskers indicate nonoutlier minima and maxima. Significance test results indicated by NS *P* > 0.05, *P < 0.05, **P < 0.01, ***P < 0.001, ****P < 0.0001. Extended statistics can be found in Supplementary Table 1. **a**, A schematic representation of the experimental timeline. After measuring baseline WGTT, mice at 30 °C were kept at 30 °C as a control or moved to 22 °C for 2 weeks. WGTT was collected again, and the change in WGTT across the 2-week period is plotted in **c**. **b**, A schematic representation of the experimental timeline. After measuring baseline WGTT, mice at 22 °C were kept at 22 °C as a control or moved to 30 °C for 2 weeks. WGTT was collected again, and the change in WGTT across the 2-week period is plotted in **d**. **c**, The change in WGTT observed in 30 °C mice after a 2-week acclimation period either remaining at 30 °C (red) or being transferred to 22 °C (blue) as determined by the difference in carmine red passage time (*N*_30°C→30°C_ = 17 (7 F, 10 M), *N*_30°C→22°C_ = 19 (5 F, 14 M); Wilcoxon test, *W* = 304.5, *P* = 6.27 × 10^−6^). **d**, The change in WGTT observed in 22 °C mice after a 2-week acclimation period either remaining at 22 °C (blue) or being transferred to 30 °C (red) as determined by the difference in carmine red passage time (*N*_22°C→22°C_ = 13 (8 F, 5 M), *N*_22°C→30°C_ = 18 (9 F, 9 M); *t*-test, *t* = −3.82, d.f. 28.9, *P* = 6.59 × 10^−4^). The mouse drawings in **a** and **b** were created with BioRender.com. Source data

We next sought to determine whether temperature-dependent differences in gut motility were determined during development or could change during adulthood. We tested whether acclimation to a new temperature for a 2-week period is sufficient to alter WGTT (Fig. 3a,b). We found that when 30 °C mice were moved to 22 °C for 2 weeks, the WGTT decreased compared to that of control mice that remained at 30 °C (Fig. 3c). In concordance with these results, when 22 °C mice were moved to 30 °C for 2 weeks, the WGTT increased compared to that of control mice that remained at 22 °C (Fig. 3d). Notably, neither control group displayed a shift in WGTT, while both experimental groups did (Supplementary Fig. 6). Our results demonstrate that differences in gut motility changed within a 2-week period, suggesting that the molecular link between housing temperature and gut motility could involve pathways that modify gut motility, such as endocrine or neuronal signaling pathways, rather than a difference in developmental trajectory.

### Temperature-dependent differences in gut motility require colon extrinsic factors

**Fig. 4:**
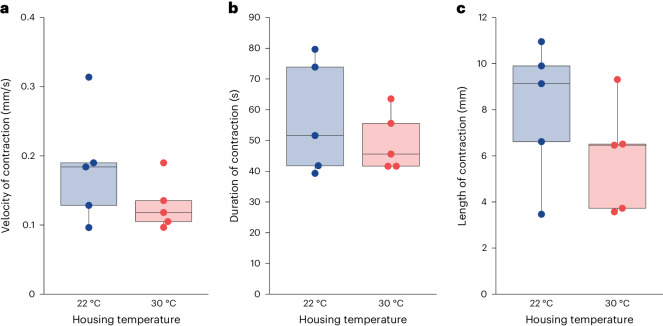
Ex vivo motility of colons from 22 °C or 30 °C mice are not statistically different. Measurements of propulsive contractions in C57BL/6 mouse colons dissected from 22 °C (blue) or 30 °C (red) mice in Krebs’ solution determined by video analysis (*N*_22°C_ = 5 (2 F, 3 M), *N*_30°C_ = 5 (3 F, 2 M). Each point is the average measurement of all annotated contractions over a 30-min period following a 10-min acclimation period to the Krebs’ solution for an individual mouse colon. Boxes indicate the upper and lower quartiles, midline represents the median and whiskers indicate nonoutlier minima and maxima. Extended statistics can be found in Supplementary Table 1. **a**, The velocity of annotated propulsive contraction waves determined via video analysis of ex vivo colon motility (*t*-test, *t* = 1.32, d.f. 5.53, *P* = 0.241). **b**, The duration of annotated propulsive contraction waves determined via video analysis of ex vivo colon motility (*t*-test, *t* = 0.820, d.f. 6.03, *P* = 0.444). **c**, The length of annotated propulsive contraction waves determined via video analysis of ex vivo colon motility (*t*-test, *t* = 1.23, d.f. 7.59, *P* = 0.257). Source data

To test whether the enteric nervous system, the neural network populating the gastrointestinal tract, or other colon-intrinsic signaling from sources such as enteroendocrine or enterochromaffin cells, drives temperature-dependent differences in gut motility, we measured the motility of colons from 22 °C and 30 °C mice in an ex vivo colonic motility assay that isolates the colon and, therefore, the enteric nervous system from extrinsic signals. Briefly, colons with cecum attached were removed and placed in Kreb’s solution. We then recorded videos and annotated propulsive contractions to measure velocity, duration and length of these motility patterns. While we observed a trend toward increased velocity, duration or length of propulsive contractions in 22 °C mouse colons compared to 30 °C mouse colons, these differences were not statistically significant (Fig. 4). We therefore concluded that the signal(s) driving temperature-dependent gut motility differences are probably colon extrinsic.

### Temperature-dependent differences in gut motility are driven by the HPA axis stress response

**Fig. 5:**
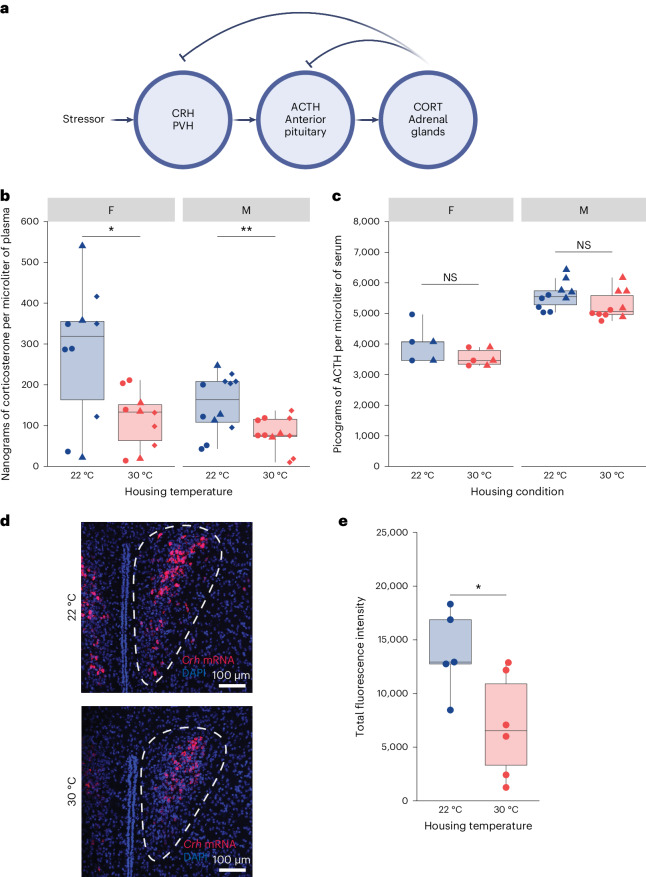
Stress hormone concentration and expression are elevated in 22 °C mice. Measurements of concentration or expression of different stress hormones. Each point represents a measurement from an individual C57BL/6 mouse. Shapes (circles, diamonds and triangles) indicate the independent experiment in which a measurement was taken. Boxes indicate the upper and lower quartiles, midline represents the median and whiskers indicate nonoutlier minima and maxima. Significance test results indicated by NS *P* > 0.05, *P < 0.05, **P < 0.01, ***P < 0.001, ****P < 0.0001. Extended statistics can be found in Supplementary Table 1. **a**, A schematic representation of the HPA axis. Stress stimulates CRH production, which stimulates ACTH production, which stimulates corticosterone (CORT) production. **b**, The plasma corticosterone concentration of 22 °C (blue) or 30 °C (red) mice measured by ELISA (*N*_F,22°C_ = 10, *N*_F,30°C_ = 10, *N*_M,22°C_ = 12, *N*_M,30°C_ = 11; *t*-tests, male 22 °C versus 30 °C: *t* = 3.07, d.f. 17.7, *P* = 0.00662; female 22 °C versus 30 °C: *t* = 2.80, d.f. 12.0, *P* = 0.0159). **c**, The plasma ACTH concentration of 22 °C (blue) or 30 °C (red) mice measured by ELISA (*N*_F,22°C_ = 5, *N*_F,30°C_ = 6, *N*_M,22°C_ = 10, *N*_M,30°C_ = 10; *t*-tests, female 22 °C versus 30 °C: *t* = 1.53, d.f. 5.34, *P* = 0.184; male 22 °C versus 30 °C: *t* = 1.68, d.f. 18.0, *P* = 0.109). **d**, *Crh* mRNA expression was measured by fluorescent in situ hybridization with RNAscope probes. The red channel indicates *Crh* and the blue DAPI channel indicates nuclei. Maximum intensity projections of representative sections of the right PVH from 22 °C and 30 °C mice shown. Sample regions of interest used for quantification drawn in dashed white lines. **e**, Quantification of *Crh* mRNA by measuring total fluorescence intensity of the PVH region of female 22 °C (blue) or 30 °C (red) mice in 20 µm sections of mouse brains (*N*_22°C_ = 5, *N*_30°C_ = 6; *t*-test, *t* = 2.63, d.f. 9.00, *P* = 0.0275). Source data

The HPA axis is a known contributor to bodily stress and is also a colon-extrinsic signal that could alter gut motility. Previous work demonstrated that administration of CRH, a central hormone in the HPA axis, in rats is sufficient to decrease small intestinal transit and increase colon transit speed^23^. This is consistent with the temperature-dependent phenotypes we observed (Fig. 1). While prior studies show a decrease in HPA axis-regulated hormone corticosterone when 22 °C mice are acclimated to 30 °C (ref. ^14^), there is a gap in research comparing stress hormone levels between mice raised at 30 °C and 22 °C. Thus, we assayed the three hormones involved in the HPA axis stress response, CRH, adrenocorticotropic hormone (ACTH) and corticosterone, in 22 °C mice and 30 °C mice (Fig. 5a). First, we tested the plasma corticosterone concentration in 22 °C mice and 30 °C mice via enzyme-linked immunosorbent assay (ELISA). We found that both male and female 22 °C mice had elevated plasma corticosterone compared to 30 °C mice (Fig. 5b). We also found a trend toward higher ACTH, the stress hormone that stimulates the production of corticosterone, in 22 °C versus 30 °C mice (Fig. 5c). Finally, we measured the expression of the peptide hormone CRH, which initiates the HPA stress response by stimulating ACTH synthesis^38^ (Fig. 5a). We performed in situ hybridization of *Crh* in the paraventricular hypothalamus (PVH) within the brains of 22 °C and 30 °C mice. We found significantly elevated expression of *Crh* in the PVH of 22 °C compared to 30 °C mice (Fig. 5d,e). Thus, both corticosterone, the primary output of the HPA stress response, and expression of *Crh*, the hormone that initiates the stress response, were significantly increased in the serum and PVH of 22 °C compared to 30 °C mice, respectively. Taken together, our results indicate that mice raised at 22 °C are more stressed than mice raised at 30 °C.

**Fig. 6:**
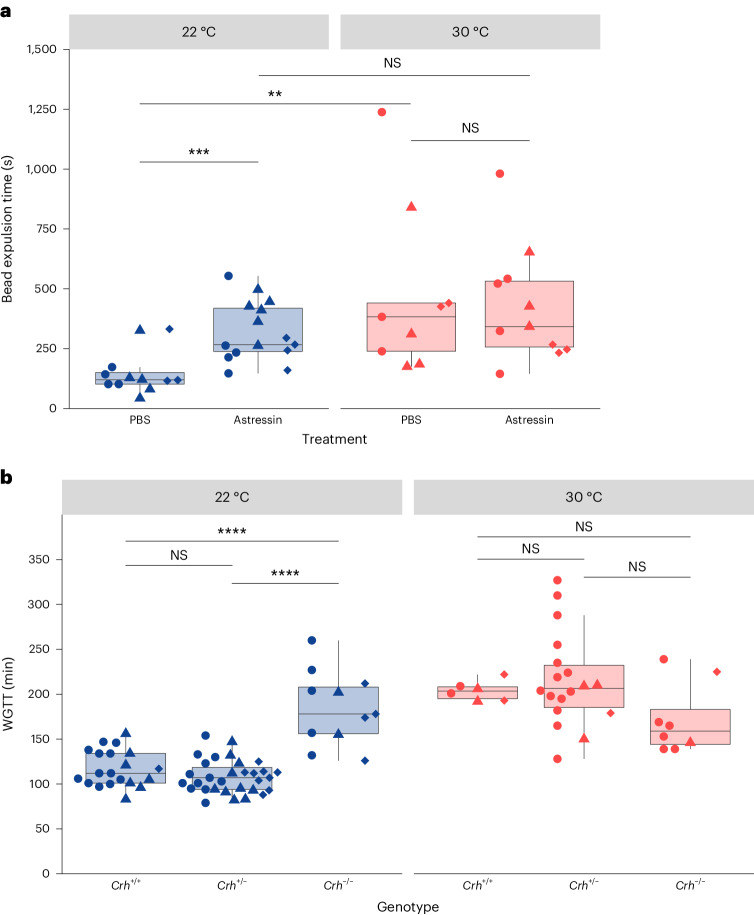
Reduced CRH activity inhibits temperature-dependent differences in gut motility. Gut motility measurements from mice with and without interventions inhibiting CRH activity. Each point represents a measurement from an individual mouse. Shapes (circles, diamonds and triangles) indicate the independent experiment in which a measurement was taken. Boxes indicate the upper and lower quartiles, midline represents the median and whiskers indicate nonoutlier minima and maxima. Significance test results indicated by NS *P* > 0.05, *P < 0.05, **P < 0.01, ***P < 0.001, ****P < 0.0001. Extended statistics can be found in Supplementary Table 1. **a**, Colon bead expulsion time for mice raised at 22 °C (blue) or 30 °C (red) was measured 30 min after injecting mice intraperitoneally with 200 μl of either treated either with PBS or 200 μg astressin per kilogram body weight in PBS (*N*_22°C,astressin_ = 15 (7 F, 8 M), *N*_22°C,PBS_ = 12 (6 F, 6 M), *N*_30°C,astressin_ = 11 (7 F, 4 M), *N*_30°C,PBS_ = 9 (5 F, 4 M); Wilcoxon tests, PBS versus astressin 22 °C: *W* = 20, *P* = 6.93 × 10^−4^; PBS 22 °C versus 30 °C: *W* = 8, *P* = 0.00122, PBS versus astressin 30 °C: *W* = 48, *P* = 0.941, astressin 22 °C versus 30 °C: *W* = 62, *P* = 0.299). **b**, WGTT of mice raised at 22 °C (blue) or 30 °C (red) was determined for mice that were homozygous wild type (*Crh*^*+/+*^), heterozygous (*Crh*^*+/*^^−^), or homozygous *Crh* deletion (*Crh*^−/−^) by a carmine red assay (*N*_22°C,*Crh*−/−_ = 11 (6 F, 5 M), *N*_22°C*,Crh*+/−_ = 31 (13 F, 18 M), *N*_22°C,*Crh+/+*_ = 20 (7 F, 13 M), *N*_30°C,*Crh*−/−_ = 8 (5 F, 3 M), *N*_30°C,*Crh+/*−_ = 18 (5 F, 13 M), *N*_30°C,*Crh+/+*_ = 6 (3 F, 3 M); Wilcoxon tests, 22 °C *Crh*^*+/+*^ versus *Crh*^−/−^, *W* = 15, *P* = 9.45 × 10^−5^; 22 °C *Crh*^*+/*^^−^ versus *Crh*^−/−^, *W* = 8.5, *P* = 3.80 × 10^−6^, 22 °C *Crh*^*+/+*^ versus *Crh*^*+*^^/−^, *W* = 394, *P* = 0.107; 30 °C *Crh*^*+/+*^ versus *Crh*^−/−^, *W* = 36, *P* = 0.137; 30 °C *Crh*^*+/*^^−^ versus *Crh*^−/−^, *W* = 106.5, *P* = 0.0588; 30 °C *Crh*^*+/+*^ versus *Crh*^*+/*^^−^, *W* = 47.5, *P* = 0.689). Source data

**Fig. 7:**
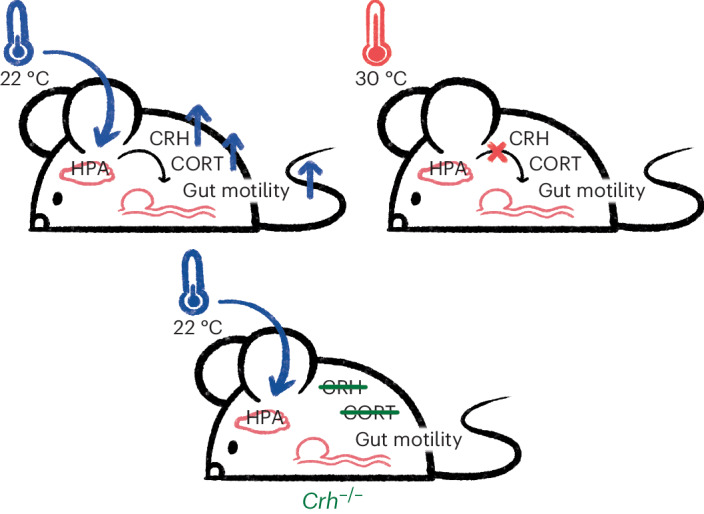
The HPA stress axis mediates temperature-dependent differences in gut motility. In our model, the HPA axis mediates increased gut motility from cold signals at 22 °C, which results in elevated stress and gut motility. At 30 °C, the absence of an environmental cold signal results in no elevation of stress hormones, and a corresponding lack of increase in gut motility. Likewise, if CRH activity is inhibited pharmacologically or genetically, mice will not experience elevated gut motility even at 22 °C because there is no stress signal to drive increased gut motility.

To evaluate the link between the HPA axis and temperature-dependent gut motility, we took pharmacological and genetic approaches. To test whether inhibition of CRH signaling would impact the temperature-dependent differences in gut motility that we saw in our mice, we injected 22 °C and 30 °C mice with the CRH inhibitor astressin. Indeed, intraperitoneal injection of astressin compared to PBS injection decreased colon transit speed in 22 °C mice but not in 30 °C mice (Fig. 6a). Moreover, astressin-treated 22 °C and 30 °C mice did not differ in colon transit time (Fig. 6a). We also took an orthogonal approach and bred CRH-deficient mice, *Crh*^−/−^ mice, at 22 °C and 30 °C (ref. ^39^). Strikingly, the genetic ablation of CRH-dependent signaling eliminated temperature-dependent differences in gut motility. Specifically, the WGTT of 22 °C *Crh*^−/−^mice was slower than the WGTT of *Crh*^*+/+*^ and *Crh*^*+/*^^−^ mice (Fig. 6b). Notably, the WGTT of 22 °C *Crh*^−/−^ mice resembled that of 30 °C mice, which did not display significant differences in WGTT between different genotypes (Fig. 6b). Taken together, these data strongly suggest that stress through the HPA axis is the primary mediator of temperature-dependent differences in gut motility (Fig. 7).

## Discussion

The effects that temperature and other environmental variables have on mouse biology have come under scrutiny for the role they have in hampering both translational outcomes and reproducibility in mouse research. Numerous researchers have proposed raising mouse facility temperatures to 30 °C, the mouse thermoneutral point, to alleviate these issues by reducing cold stress experienced at standard facility temperatures of 22 °C (refs. ^2,3,13^). However, scientists lack a detailed catalog of the impact of this temperature increase on mouse biology and what mouse models of disease or physiology would be influenced by such a shift. A handful of studies have explored the effects of thermoneutral environments on the mouse gastrointestinal system, but there is no research on how basic, yet critical, aspects of gut physiology such as gut motility change with ambient temperature^14,29,30^. To address this gap in knowledge, we examined how the gut physiology of mice raised at 22 °C differs from that of mice raised at 30 °C.

We measured different aspects of gut motility in 22 °C and 30 °C mice and found that 22 °C mice displayed WGTTs roughly twice as fast as those of 30 °C mice. This was primarily driven by a threefold increase in the speed of colonic transit, as opposed to a 10% decrease in the speed of small intestinal transit (Fig. 1). These results indicate that proposed shifts of mouse facilities from 22 °C to 30 °C will alter gut motility, potentially affecting other phenotypes of interest. For example, using thermoneutral mice instead of cold-stressed mice may result in increases in the bioavailability of drugs delivered orally or rectally, which could produce large swings in metrics like toxic dose or effective dose^40^. In addition to the shifts in gut transit time, we also observed significant compositional differences at the species level in the microbiota of animals reared at 22 °C and 30 °C (Fig. 2). Other studies have also shown that, compared to mice raised at 30 °C or acclimated to 30 °C, the gut microbiota composition of 22 °C mice differs, albeit at a genus level^14,29,30^. Notably, Giles et al. and Hylander et al. find a reduction in the abundance of the genus *Akkermansia* at 22 °C (refs. ^14,29^), which is concordant with our finding that *A. muciniphila* is reduced at 22 °C (Fig. 2d). Additionally, our findings in mice also agree with previous findings that a low abundance of *A. muciniphila* is associated with faster gut motility in humans^32–34^. It may be that elevated gut motility at 22 °C compared to 30 °C therefore explains temperature-dependent compositional differences that previous groups have observed^14,29,30^. Given the well-described causal relationship between the gut microbiota and various disease-related animal model outcomes, it is possible that housing temperature may also alter the outcomes of experiments that use mouse models of human disease. The functional differences between 22 °C and 30 °C mouse gut microbiota remain an open question, and future studies defining these could inform what other temperature-dependent changes in host physiology may be mediated by the microbiota. Likewise, there is keen interest in using thermoneutral animals to improve the translatability of immunology research done in mice^2,13,41^. Researchers seeking to study gut immunity may therefore need to account for the effects of decreased gut motility, such as increased susceptibility to enteric pathogens, in their models of study if they opt to use thermoneutral mice^26,42^. At present, it is unclear if alterations in ambient temperature independently impact immune function and the microbiota, or if impacts on one of these parameters affect the other. Additional research will be required to investigate these hypotheses. However, we speculate that numerous gut phenotypes may differ at 22 °C and 30 °C, given the breadth of consequences of altered gut motility.

We next sought to characterize the mechanistic drivers of temperature-dependent differences in gut motility. Our results indicate that the gut microbiota (Fig. 2) and differential food intake (Supplementary Fig. 4) are unlikely to drive the temperature-dependent phenotypes that we observed. While certain studies report increased food consumption at 22 °C compared to 30 °C in mice^13,36^, it is critical to highlight that many employ singly housed mice acclimated to a new temperature, not group-housed mice reared at 22 °C or 30 °C, as in our study. Maher et al. showed that group housing, while not entirely compensating for cooler temperatures, results in comparable food intake between group-housed mice at 20 °C and 30 °C mice^37^, aligning with our results. In addition to the slight amelioration of cold stress by group housing, another interesting possibility for how 22 °C mice can maintain similar body weights and increases in body weight over time (Supplementary Fig. 5) could be provided in the findings published by Chevalier et al.^43^. In this study, they demonstrate that a 31-day period of cold exposure led to a reduction in *A. muciniphila* abundance, which is consistent with our findings (Fig. 2d). They also found that the reduction in *A. muciniphila* abundance at lower ambient temperatures drove lengthening of the SI and increased villus height, allowing mice housed at a colder ambient temperature to absorb more nutrients^43^. This could explain, in part, why we did not observe a statistically significant increase in food consumption by mice housed at 22 °C. This, coupled with the compensation for cold provided by group housing, may explain why we only observed a trend toward higher food intake in 22 °C mice. We therefore concluded that differential food intake did not drive temperature-dependent differences in gut motility.

Having concluded that variations in food consumption are not primarily responsible for temperature-dependent differences in WGTT, we explored discrepancies in HPA stress hormones, which are known to influence WGTT. We found that 22 °C mice have elevated plasma corticosterone concentrations and expression of *Crh*, two hormones involved in the HPA axis stress response. Our data resemble previous results showing that increased plasma corticosterone and *Crh* expression occurred in neonatal rats and increased corticosterone occurred in adult mice exposed to colder temperatures^14,44^. Collectively, the cold-induced HPA axis stress response seems generalizable across rodents and studies. Interestingly, we noticed a previously documented sex-dependent difference in corticosterone levels in 22 °C mice^45,46^, but not an associated sex-dependent difference in WGTT in 22 °C mice. This could mean that the effects of stress on gut motility are already saturated at the relatively lower stress levels observed in males at 22 °C, or that the effects of stress on changes in gut motility are dampened in female mice and require a higher stress level to achieve the same effect as in males. Additionally, we did not observe a sex-dependent difference in stress levels for mice housed at 30 °C, which could lend support to the argument that, in some experimental models, housing animals at 30 °C could improve reproducibility. Speculatively, we hypothesize that the smaller body mass of female mice makes them more susceptible to cold stress at 22 °C, but housing at thermoneutrality eliminates this source of stress, thereby ablating the sex-dependent differences in corticosterone. An interesting question that stems from these observations is whether certain models of stress, such as social isolation, should be applied at 30 °C instead of 22 °C. This not only introduces the stress of isolation but also removes the slight reduction of cold stress provided by group housing^37^, thus potentially confounding one source of stress with another. More broadly, studying how thermal stress, or lack thereof, impacts the outcomes of stress-related studies could better inform efforts to improve the translational capacity of research.

Finally, we tested whether elevated stress at 22 °C was responsible for gut motility differences by taking both pharmacological and genetic approaches. Depleting CRH with astressin or genetic deletion of *Crh* in 30 °C mice had no effect on gut motility, but reduced motility in 22 °C mice to match that of 30 °C mice. While we did not test the effects of stress induction in this study, a previous study found that administration of CRH, or restraint stress, to rats results in no change in gastric emptying, a decrease in small intestinal motility and an increase in colonic motility. This matches the motility patterns we see in 22 °C relative to 30 °C mice, further supporting our model^23^. The HPA stress response affects many biological functions, so it is difficult to say whether there is a direct link between elevated HPA activity in 22 °C mice and faster gut transit, or whether other downstream effects of corticosterone mediate motility changes^38^. However, since removing CRH is sufficient to make 22 °C mice phenocopy 30 °C mice but does not affect gut motility in 30 °C mice, we conclude that the HPA stress response is the primary driver of temperature-dependent differences in gut motility (Fig. 7). We also highlight how this finding supports our earlier conclusion that food intake differences do not drive temperature-dependent differences in gut motility. *Crh*^−/−^ mice are known to consume the same amount of food as *Crh*^*+/+*^ mice^47^, but despite this, we observe drastic differences in the WGTT of mice of these two genotypes at 22 °C.

Although we show that elevated stress mediates increased gut motility at 22 °C versus 30 °C, there are still unanswered questions and limitations to our study. For example, we do not yet know what differences in gut motility patterns mediate increased motility at 22 °C in vivo. Since we see the strongest effect of ambient temperature on colon motility, we speculate that giant migrating contractions, which primarily move large boluses of material along the colon, are elevated at 22 °C (ref. ^48^). If this is the case, future studies using mice at 30 °C could lead to better translational models for humans, as the increased giant migrating contraction frequency in mice is one of the primary differences between mouse and human colon motility^48^. An additional unanswered question is whether the type and timing of stress affect gut motility. For example, Schneider et al. found that applying restraint stress to 22 °C mice for 3 h a day over a week decreased WGTT^24^. By contrast, another study found that applying restraint stress or administering CRH for 30 min to 22 °C mice resulted in a large increase in colon motility^23^. Our findings suggest that chronic cold stress resembles the more acute stress response to gut motility that Williams et al. observed^23^, but whether it drives the differences through the same intermediate pathways downstream requires further study. One limitation in our research concerning stress is that, due to logistical constraints, 30 °C mice experienced brief exposure to the 22 °C temperature of the procedure room. Although they were promptly returned to 30 °C during waiting periods for each procedure, except for the colon transit time assay, this brief exposure could introduce an unpredictable stressor. Moreover, it is known that thermoneutral housing can impact mouse metabolism^7^, potentially influencing the regulation of gastrointestinal motility. However, measuring metabolic rates requires single-housed animals, while our study employs group-housed animals to accurately represent typical vivarium conditions. Consequently, investigating how metabolic changes at 30 °C could contribute to altered gastrointestinal motility is an important avenue for future research. Additionally, we did not explore whether differential water intake could account for variations in WGTT, which warrants further investigation. Finally, while antibiotic treatment resulted in a significant decrease in fecal microbial load (Fig. 3), there remains a possibility that certain uncultured taxa critical for WGTT may have survived the antibiotic treatment at minimal levels. Further study using germ-free mice could conclusively rule out this possibility.

While our study demonstrates the temperature dependence of gut motility and HPA stress, the specific signaling pathways upstream that trigger these responses remain unknown. Cold-sensing pathways, such as those mediated by the cold temperature receptor TRPA1 (ref. ^49^) might transduce signals at 22 °C but not at 30 °C. Increased stress at lower temperatures could also result from social stressors, as lower temperatures can affect certain social behaviors such as huddling^50^. In summary, future studies elucidating the pathways through which different forms of stress influence gut motility could offer valuable insights and contribute to the development of methods for treating stress-derived gastrointestinal motility disorders.

Viewed through the lens of animal research, our discoveries highlight temperature as a critical abiotic factor that could introduce reproducibility challenges. Different institutions adhere to diverse guidelines for the recommended temperatures for housing mice, ranging from 20 °C to 26 °C (ref. ^51^), to 18 °C to 23 °C (ref. ^52^), to 20 °C to 24 °C (ref. ^53^). Even within these recommended ranges, there is a broad spectrum of acceptable temperatures. Our study demonstrates that an 8 °C variance is sufficient to introduce a twofold change in gut motility, accompanied by changes in the composition of the gut microbiota. However, we do not yet know whether the relationship between gut motility and temperature change is linear or if there exists a critical ‘threshold’ temperature between 22 °C and 30 °C, beyond which motility and microbiota composition suddenly change. Understanding this will guide how closely animal facility temperatures must be regulated and whether studies conducted across different facilities are comparable.

Additionally, it is critical to acknowledge that temperature represents just the metaphorical tip of the iceberg when considering how animal facilities can best be regulated to improve translation. Temperature interacts with other environmental variables, such as relative humidity, which tends to be a relatively uncontrolled factor in vivaria, resulting in varied effects on energy expenditure^54^. Therefore, a comprehensive effort to study how various environmental factors impact mouse biology, while challenging, is necessary to inform future best practices.

The effects of fluctuating temperatures on animal physiology are becoming increasingly relevant in various settings. We hope our research prompts further dialogue regarding the consequences of rising temperatures on animal physiology, whether in the context of the translational value of mouse research or as potential sources of variability across studies. Furthermore, we contend that gaining a more comprehensive understanding of how thermal stress and other stressors influence the gut–brain axis will enrich our understanding of illnesses related to stress. Ultimately, achieving a deeper understanding of how environmental factors affect gut biology will prove critical for advancements in each of these domains.

## Methods

### Animal strains and husbandry

Breeder pairs of C57BL/6NJ (RRID:IMSR_JAX:005304) and 129×1/SvJ (RRID:IMSR_JAX:000691) mice were obtained from The Jackson Laboratories. Breeder pairs of C57BL/6J-*Crh*^*+/*^^−^ mice were generously donated by Dr. Joseph Majzoub at Boston Children’s Hospital; the strain generation is detailed in Zhang et al.^39^. To establish the breeding colonies, 6–8-week-old littermates were divided between 22 °C and 30 °C specific pathogen-free conditions in Innovive filter-top cages that were changed every 2 weeks by veterinary or research personnel. The breeder mice were given a week to acclimate to 30 °C before being set up in breeder pairs, with the first litter being born at least 3 weeks later. Sterile chlorinated water (Innovive M-WB-300C) and food (Envigo Teklad Global 18% Protein Rodent Diet) were provided ad libitum. Additionally, mice were supplemented with Enviro-dri and Alpha-dri bedding. All experiments were conducted in the procedure room after animals were moved to incubators in the same procedure room. Animals were given at least 10 days to acclimate to a new environment before experimentation. Genotypes of C57BL/6J-*Crh*^*+/*^^−^ mice were verified via PCR as described by Zhang et al.^39^. The offspring were kept in single-sex cages and housed under the same conditions as the breeder pairs, with group sizes ranging between two and five mice. The offspring were housed in rodent incubators in the same room that were set to either 22 °C or 30 °C. All experiments were conducted with adult mice (10–18 weeks of age). All experiments were started in the morning between 9:00 and 12:00, corresponding to ZT2 and ZT5. All experiments, unless otherwise stated, used a mix of male and female mice (exact numbers stated in figure legends). Experimenters were not blinded to treatment groups. All experiments were conducted according to Stanford APLAC standards under protocol number 12826.

### Shotgun-metagenomic sequencing and analysis

Fecal pellets were collected from animals and immediately frozen on dry ice before storage at −80 °C. Subsequently, DNA was extracted from pellets using the QIAamp Fast DNA stool mini kit according to manufacturer’s instructions with an additional bead-beating step (5 min with 1 mm zirconium beads) after samples were placed in InhibitEx buffer. Sequencing libraries were prepared using the Illumina Nextera XT DNA Library Preparation Kit and were subsequently sequenced on the Illumina Hiseq 4000 platform. Reads were processed using the Kraken2 workflow found at https://github.com/bhattlab/kraken2_classification/tree/master^55^. In brief, reads were classified using Kraken2 and Bracken with reference genomes from the Mouse Gastrointestinal Bacteria Catalogue Version r1.g1021 (ref. ^31^). Subsequently, postprocessing analyses of beta diversity, alpha diversity and species enrichment were also performed in R, the code for which can be found in the same GitHub repository as above.

### WGTT assay

Animals were fasted overnight for 16 h. Animals were orally gavaged with 300 μl of 6% carmine (Sigma C1022, CAS 1390-65-4) suspended in 0.5% methylcellulose (Sigma, M0387), then single-housed in cages and provided with food and water. Cages were checked every 15 min without moving or disturbing the animals for the presence of carmine in fecal pellets. The time between gavage and the first presence of a red fecal pellet is noted as WGTT.

### Temperature-swap assay

The WGTT assay described above was first performed. Immediately after the assay, control mice were placed back in their original incubators, while experimental mice were moved to an incubator at a different temperature. All mice acclimated for 2 weeks before the WGTT assay was performed again. The change in transit time between the second and first WGTT assays for each individual mouse was calculated by subtracting the first time from the second. Cages were assigned to the experimental and control groups randomly.

### Gastric emptying and small intestinal transit assay

Assays of gastric emptying and small intestinal transit were adapted from De Lisle et al.^56^. Animals were fasted overnight for 16 h to reduce variability. Animals were orally gavaged with 100 μl of 25 ng/μl FITC–dextran 70 kDa in 0.5% methylcellulose (Sigma, M0387; CAS 9004-67-5) and placed back in cages. After 30 min, animals were euthanized and stomach and SIs were collected, with the SI being divided into eight equal length segments. Each piece of tissue was placed in a 15 ml conical tube with 2 ml of PBS and homogenized. One milliliter of each homogenized tube was centrifuged for 5 min at 10,000× relative centrifugal field (RCF). The supernatant was diluted 1:10, then 200 μl was loaded onto an Agilent BioTek Synergy HTX Multimode Reader to quantify fluorescence. Gastric emptying of each animal was calculated by dividing the fluorescence of the stomach supernatant by the sum fluorescence of the stomach and all SI segments from an individual. Small intestinal transit was determined by calculating the geometric mean of the fluorescence as $\scriptstyle({\sum }_{n=1}^{8}{{\mathrm{Segment}}\; n}\times {{\mathrm{Fluorescence}}})/{{\mathrm{Total}}\; {\mathrm{small}}\; {\mathrm{intestinal}}\; {\mathrm{fluorescence}}}.$

Leading-edge small intestinal transit was calculated by gavaging mice with 200 μl of 10% activated charcoal in 0.5% methylcellulose (Sigma, M0387; CAS 9004-67-5). After 30 min, animals were euthanized, and the distance traveled through the SI by the leading edge of the charcoal meal bolus was divided by the total length of the SI.

### Colon bead expulsion assay

Colonic motility was assessed according to Spear et al.^57^. In brief, animals were fasted overnight for 16 h to reduce variability. Animals were anesthetized with isoflurane, and then a 3 mm glass bead lubricated with 10% glycerol was gently inserted 2 cm up the rectum. Animals were then placed in an empty cage and monitored until they expelled the glass bead. If the bead could not be inserted for the full 2 cm due to the presence of a fecal pellet or if the bead was immediately accompanied by a fecal pellet when expelled, the assay was performed again following the excretion of the pellet. The difference between the time of insertion and the time of expulsion of the glass bead was recorded as a measure of colon motility.

### Astressin administration

Mice were handled according to the colon bead expulsion assay described above. Before fasting, mice were weighed to determine the necessary dose. The next morning, mice were injected intraperitoneally with either a dose of 200 μg astressin per kilogram body weight in 200 μl of phosphate-buffered saline (PBS) or a control solution of PBS (MedChem Express, HY-P0257), then placed back into their original cages. After 30 min, the colon bead expulsion assay was performed. Animals were assigned to treatment groups in a stratified manner, such that each cage had at least one animal in each of the control and treatment groups.

### Ex vivo colon motility

Ex vivo colon motility was measured as described in Swaminathan et al. and Robinson et al.^58,59^. In brief, colons dissected from mice were pinned at the cecum and distal colon in an organ bath of circulating Krebs’ solution (NaCl, 120.9 mM; KCl, 5.9 mM; NaHCO_3_, 25.0 mM; monobasic NaH_2_PO_4_, 1.2 mM; CaCl_2_, 3.3 mM; MgCl_2_•6H_2_O, 1.2 mM; d-glucose, 11.1 mM) kept at 37 °C and saturated with 5% CO_2_, 95% O_2_. Colons were allowed to acclimate to the solution for 10 min. Colonic contraction activity was recorded with a high-resolution monochromatic firewire industrial camera (The Imaging Source, DMK41AF02) and subsequently analyzed using Scribble 2.0 and the Matlab 2012a plugin Analyse 2.0 (ref. ^58^).

### Average food intake measurements

Food baskets in cages of group-housed mice were weighed at 17:00 (10 ZT) daily over a 3-day period. The average food consumed per mouse per day in each cage was calculated using the formula below. In brief, total food consumed over the 3-day period in each cage was normalized to the number of mice per cage per day as

$$\begin{array}{l}({{\rm{D}}}_{3}\,{\rm{food}}\,{\rm{weight}}-{{\rm{D}}}_{0}\,{\rm{food}}\,{\rm{weight}})/(3\,{\rm{days}}\,\times \,{\rm{Number}}\,{\rm{of}}\,{\rm{mice}}\,{\rm{per}}\,{\rm{cage}})\\={{\rm{Average}}\; {\rm{food}}\; {\rm{consumed}}\; {\rm{per}}\; {\rm{mouse}}\; {\rm{per}}\; {\rm{day}}}.\end{array}$$

### Antibiotic treatment

Mice were provided with either a vehicle-only solution of 40 g/l Kool-Aid Grape or a vehicle plus antibiotic solution of 40 g/l Kool-Aid Grape Drink Mix Sugar Sweetened, 1 g/l of vancomycin hydrochloride (Gold Biotechnology, V-200-5, CAS 1404-93-9), 1 g/l of ampicillin sodium salt (Fisher Scientific, BP1760, CAS 69-52-3), 1 g/l of neomycin sulfate (Enzo, ALX-380-035-G025) and 0.5 g/l of metronidazole (Spectrum Chemical, M1511, CAS 443-48-1) ad libitum in drinking water for 2 weeks before performing the WGTT assay. Solutions were replaced with fresh stock each week. Cages were assigned to the vehicle or antibiotic treatment randomly. Microbiome depletion was verified by adapting the protocols used by Amorim et al.^60^ and Reikvam et al.^61^. In brief, fecal pellets were collected and homogenized in 500 μl of PBS. One-hundred microliters of a 1:1,000 dilution of the homogenate in PBS was plated on tryptic soy agar with 5% sheep’s blood plates. The plating method we employed, as described by Amorim et al.^60^, yields either a complete covering of the plate or a lack of growth as the outcome under aerobic and anaerobic conditions. Employing the identical depletion cocktail, we replicated the observed outcomes, detecting as low as 10^4^ colony-forming units per milliliter of PBS in the original fecal suspension undiluted in 1 ml of PBS. Given a median fecal sample weight of 44 mg, this corresponds approximately to a detection limit of 227 colony-forming units per milligram of feces. Further, to verify that DNA content in feces dropped in antibiotic-treated animals as has been previously observed^60^, DNA from feces was extracted using the same method as in the shotgun-metagenomic sequencing section and concentration was measured using the Qubit dsDNA Broad Range Quantification Assay Kit (ThermoFisher, Q32850).

### Corticosterone and ACTH ELISA

Plasma was collected in the morning at 10:00 (3 ZT) via cardiac puncture immediately following euthanasia by carbon dioxide. In essence, we followed the procedure described by Powell et al.^62^, as they show that, among the different euthanasia methods tested, carbon dioxide euthanasia produced the lowest spike in serum corticosterone. Blood was collected into Eppendorf tubes containing 10 μl of 5 U/μl heparin sodium salt from porcine intestinal mucosa (Sigma, H3149). Samples were centrifuged at 2,000× RCF for 10 min, and plasma was collected, aliquoted and stored at −80 °C. Plasma was then used in either the Mouse/Rat ACTH SimpleStep ELISA Kit (ab263880, Abcam) or the DetectX Corticosterone Enzyme Immunoassay Kit (K014-H1) to measure ACTH and corticosterone, respectively, according to the manufacturers’ instructions. Absorbances were read on an Agilent BioTek Synergy HTX Multimode Reader.

### Serotonin ELISA

Feces were collected from mice and immediately frozen on dry ice before storage at −80 °C. Feces were added to 250 μl of 0.1% ascorbic acid in PBS in bead-beating tubes loaded with ~20–25 1-mm-diameter zirconium beads. Samples were homogenized for 30 s using a bead beater and centrifuged at 10,000× RCF for 10 min. The supernatant was then used in the DLD Serotonin ELISA (REF EA602/96). Absorbances were read on an Agilent BioTek Synergy HTX Multimode Reader.

### CRH FISH and imaging

To spatially detect RNA, RNAScope Fluorescent Multiplex Reagent Kit v2 (Advanced Cell Diagnostics, Catalog # 323136) was used according to the manufacturer’s recommended protocol. Briefly, female mice were deeply anesthetized and decapitated, and the brain was quickly dissected. The brain was embedded in OCT embedding gel (Tissue-Tek, Sakura Finetek) and snap frozen in an ethanol dry ice bath. The brain was sliced into 20 µm sections in the cryostat and mounted on Superfrost Plus slides. PVH-containing slides were fixed in 4% paraformaldehyde for 1 h and 30 min at room temperature, then dehydrated using 50%, 70% and 100% ethanol for 5 min each. A protease solution (pre-treatment IV, Advanced Cell Diagnostics) was applied to slices for 5 min at room temperature and washed with PBS. Next, target probes were applied, and slides were incubated in a hybridization oven at 40 °C for 2 h, followed by amplification steps at 40 °C (Amp1 30 min, Amp2 30 min, Amp3 15 min, RNAscope Multiplex FL v2 HRP-C1 15 min, C1 probe, RNAscope Multiplex FL v2 HRP blocker). Slides were then covered with DAPI Fluoromount-G (Southern Biotech). RNAScope *Mus musculus* Crh mRNA (catalog no. 31609) and TSA Vivid Fluorophore 650 (catalog no. 32327) were used. All images were taken using a Zeiss LSM 700 confocal microscope with Zen 2009 software on a 20× objective lens in 1 μm increments through the tissue section in 660 and 488 channels. Imaging parameters were held constant for all brain sections. Images were analyzed using the image analysis software CellProfiler (Broad Institute). In brief, we identified the PVH, applied a lower threshold to remove background noise and quantified fluorescence intensity within the region of interest in the 660 channel.

### Statistical Analysis

Statistical tests were performed in R version 4.2.1. All statistical analyses were two-sided, and statistical significance was assessed at *α* = 0.05. Statistical significance was assessed using the Stats (version 4.2.1) package using default parameters and commands. Student’s *t*-tests were performed if the data were normal according to a Shapiro–Wilk test of normality. Otherwise, a Wilcoxon rank-sum test was performed. No custom code was used. Statistics are reported in Supplementary Table 1 under tabs corresponding to each respective figure. For all experiments, animals were excluded from analyses if they required euthanasia before completion of the experiment due to signs of distress or if samples collected from animals did not meet manufacturer guidelines (for example, plasma showed signs of hemolysis for ELISA assays).

### Reporting summary

Further information on research design is available in the Nature Portfolio Reporting Summary linked to this article.

## Online content

Any methods, additional references, Nature Portfolio reporting summaries, source data, extended data, supplementary information, acknowledgements, peer review information; details of author contributions and competing interests; and statements of data and code availability are available at 10.1038/s41684-024-01376-5.

### Supplementary information

Supplementary Information Supplementary Figs. 1–6.

Reporting Summary

Supplementary Data Statistical source data for Supplementary Figs. 1–6.

Supplementary Data Statistical summary data for all figures.

### Source data

Source Data Fig. 1 Statistical source data for Fig. 1.

Source Data Fig. 2 Statistical source data for Fig. 2.

Source Data Fig. 3 Statistical source data for Fig. 3.

Source Data Fig. 4 Statistical source data for Fig. 4.

Source Data Fig. 5 Statistical source data for Fig. 5.

Source Data Fig. 6 Statistical source data for Fig. 6.

## Data Availability

The metagenomics sequencing results that support the findings of this study have been deposited on Sequence Read Archive under the BioProject accession PRJNA1069241. The microscopy files from Fig. 5d and videos from Fig. 4 have been deposited on the Stanford Digital Repository at 10.25740/sc811vz1270. Source data are provided with this paper.
