# Supplementary material for: Temperature-dependent differences in mouse gut motility are mediated by stress
Source: Lab Anim (NY). 2024 May 28;53(6):148–59. doi: 10.1038/s41684-024-01376-5 (PMC11147774; doi:10.1038/s41684-024-01376-5)
Supplement: Supplementary file 1 — Supplementary Figs. 1–6. [file 41684_2024_1376_MOESM1_ESM.pdf]

lab animal

https://doi.org/10.1038/s41684-024-01376-5

Temperature-dependent differences in mouse gut motility are mediated by stress

In the format provided by the authors and unedited

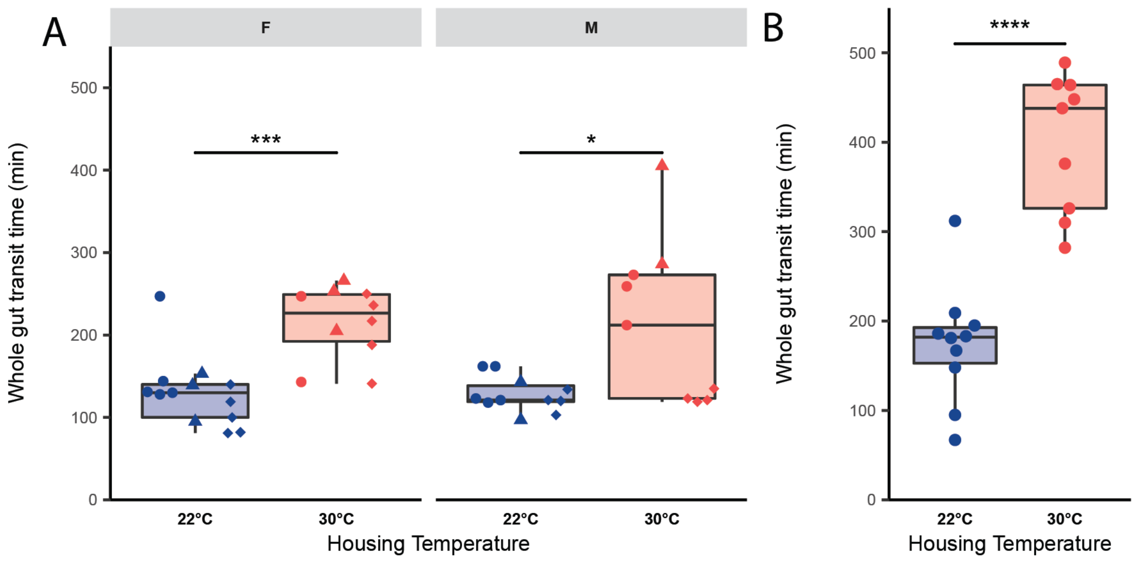

Figure S1. Temperature-dependent differences in gut motility are independent of sex and genetic background of mice. Each point represents a measurement from an individual C57BL/6 mouse. Boxes indicate the upper and lower quartiles, midline represents the median, and whiskers indicate non-outlier minima and maxima. Significance test results indicated by * $p < 0.05$, ** $p < 0.01$, *** $p < 0.001$, **** $p < 0.0001$. Extended statistics can be found in Supplementary Table 1.

S1A) The same data as Fig 1A plotted by sex, showing the whole gut transit time of male and female 22°C and 30°C mice determined by the time difference between oral gavage and defecation of carmine red dye. ($N_{22°C,\,F} = 13$, $N_{30°C,\,F} = 10$, $N_{22°C,\,M} = 11$, $N_{30°C,\,M} = 9$; Wilcoxon tests, Females: W = 21.5, p = 8.09E-4; Males: W = 10.5, p = 0.0363). Each point represents a measurement from a separate, individual mouse. Shapes (●, ◆, ▲) indicate the independent experiment in which a measurement was taken.

S1B) Whole gut transit time of 129x1/SvJ mice raised at 22°C (blue) or 30°C (red) was determined by the time difference between oral gavage and defecation of carmine red dye. Each point represents ($N_{22°C} = 10$ (5 F, 5 M), $N_{30°C} = 9$ (4 F, 5 M); T-test, t = -6.78, df = 15.9, p = 4.60E-6).

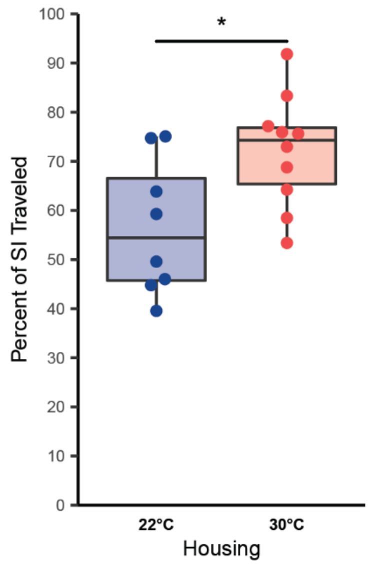

Figure S2. Small intestinal motility is faster in 30°C mice when measured by leading edge transit. Small intestinal motility of 22°C (blue) and 30°C (red) C57BL/6 mice as determined by percent of the small intestine traversed by the leading edge of a bolus of activated charcoal thirty minutes after gavage ($N_{30°C}$ = 10 (6 F, 4 M), $N_{22°C}$ = 8 (4 F, 4 M); T-test, df = 13.7, t = -2.57, p = 0.0226). Each point represents a measurement from an individual mouse. Boxes indicate the upper and lower quartiles, midline represents the median, and whiskers indicate non-outlier minima and maxima. Significance test results indicated by * $p < 0.05$, ** $p < 0.01$, *** $p < 0.001$, **** $p < 0.0001$. Extended statistics can be found in Supplementary Table 1.

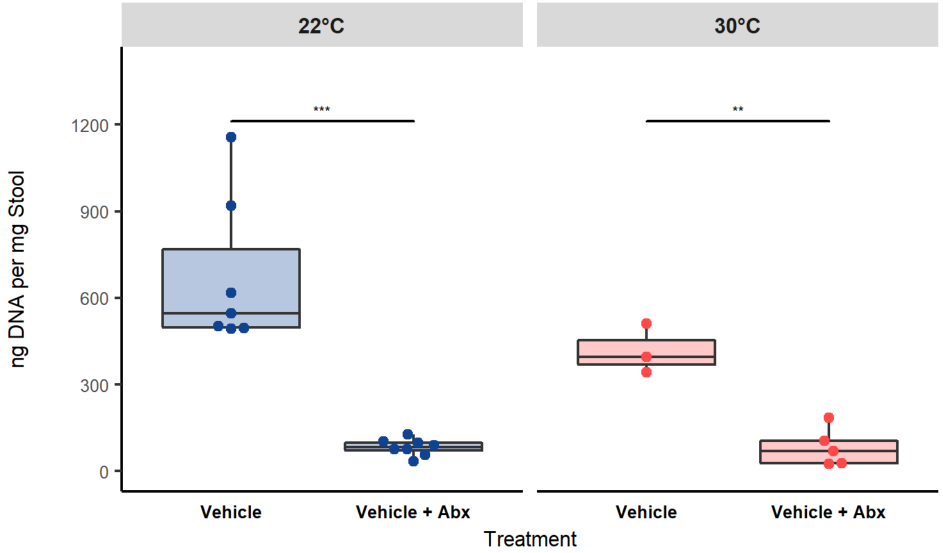

Figure S3. DNA concentration extracted from stool in untreated animals versus antibiotic-treated mice. Concentration of DNA extracted from of stool samples collected from individual 22°C (blue) or 30°C (red) C57BL/6 mice treated with a vehicle solution or vehicle plus antibiotic solution of vancomycin, neomycin, ampicillin, and metronidazole ($N_{22°C,Vehicle}$ = 7 (7 F), $N_{22°C,Vehicle+Abx}$ = 8 (4 F, 4 M), $N_{30°C,Vehicle}$ = 3 (3 F), $N_{22°C,Vehicle}$ = 5 (3 F, 2 M); T-tests, 22°C: $p < 0.001$, 30°C: $p = 0.007$). Each point represents a measurement from an individual mouse. Boxes indicate the upper and lower quartiles, midline represents the median, and whiskers indicate non-outlier minima and maxima. Significance test results indicated by NS $p > 0.05$, * $p < 0.05$, ** $p < 0.01$, *** $p < 0.001$, **** $p < 0.0001$. Extended statistics can be found in Supplementary Table 1.

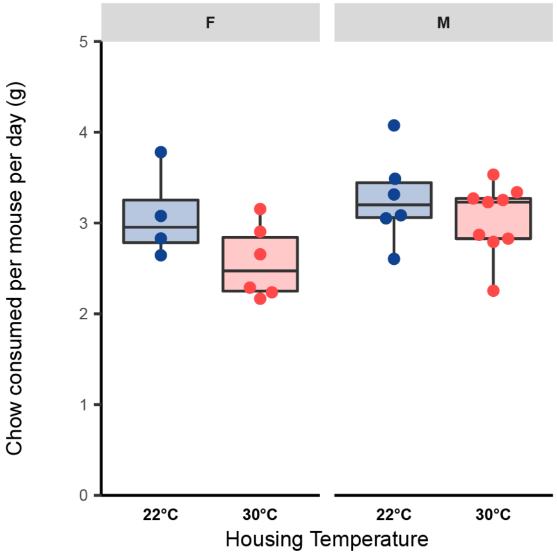

Figure S4. Cohoused adult 22°C mice do not consume significantly more food than 30°C mice. Each point represents the average chow consumed per mouse per day over a three-day period by a group of mice housed in a single cage. Data are stratified by sex of the mice ($N_{22°C, F}$ = 4 cages, $N_{30°C, F}$ = 6 cages, $N_{22°C, M}$ = 6 cages, $N_{30°C, M}$ = 9 cages; T-tests, Female 22°C vs 30°C: t = 1.73, df = 5.56, p = 0.139; Male 22°C vs 30°C: t = 0.951, df = 9.05, p = 0.366). Boxes indicate the upper and lower quartiles, midline represents the median, and whiskers indicate non-outlier minima and maxima. Extended statistics can be found in Supplementary Table 1.

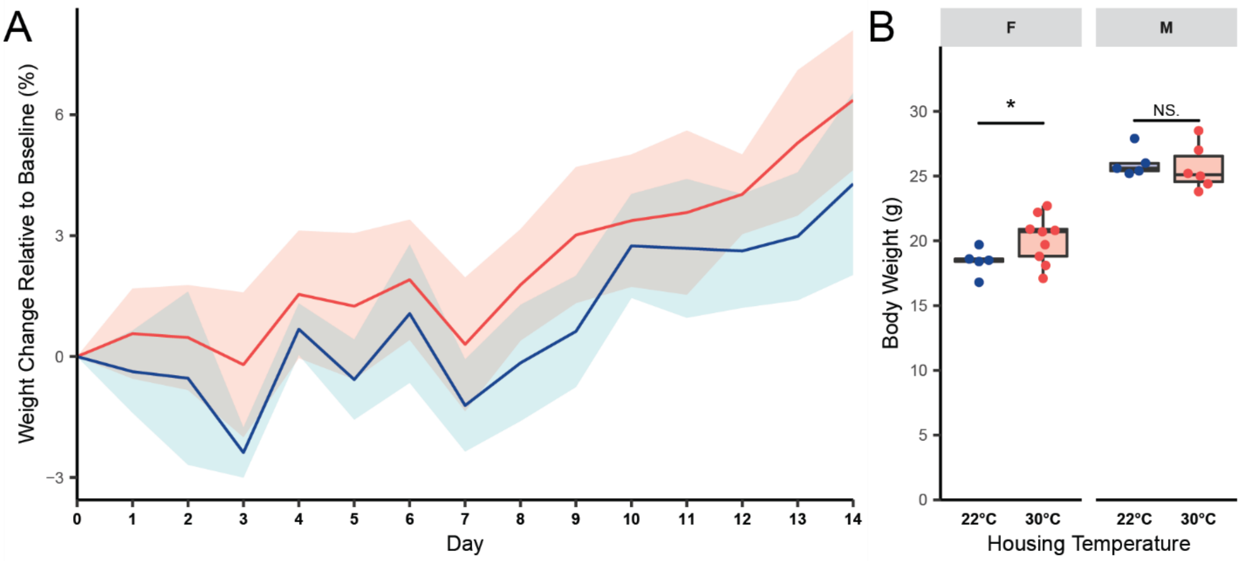

Figure S5. There is no difference in weight change for mice housed at 30°C and 22°C over a two-week period but body weight of female adult mice differs slightly. Individual adult 22°C (blue) and 30°C (red) C57BL/6 mice were weighed over a two-week period. Extended statistics can be found in Supplementary Table 1.

S4A) Change in weight relative to baseline over two weeks of adult 22°C (red) and 30°C (blue) mice. Solid lines represent mean change in relative weight and shaded ribbons represent 95% confidence intervals. ($N_{22°C}$ = 5 (2 F, 3 M), $N_{30°C}$ = 7 (4 F, 3 M))

S4B) Body weight of individual adult 22°C (blue) and 30°C (red) mice of both sexes. Data are stratified by sex. Each point represents a measurement from an individual mouse. Boxes indicate the upper and lower quartiles, midline represents the median, and whiskers indicate non-outlier minima and maxima. Significance test results indicated by NS $p > 0.05$, * $p < 0.05$, ** $p < 0.01$, *** $p < 0.001$, **** $p < 0.0001$. ($N_{22°C, F}$ = 5, $N_{22°C, M}$ = 5, $N_{30°C, F}$ = 9, $N_{30°C, M}$ = 6; T-tests, Female 22°C vs 30°C, t = -2.21, df = 12, p = 0.0470; Male 22°C vs 30°C, t = 0.425, df = 8.43, p = 0.681).

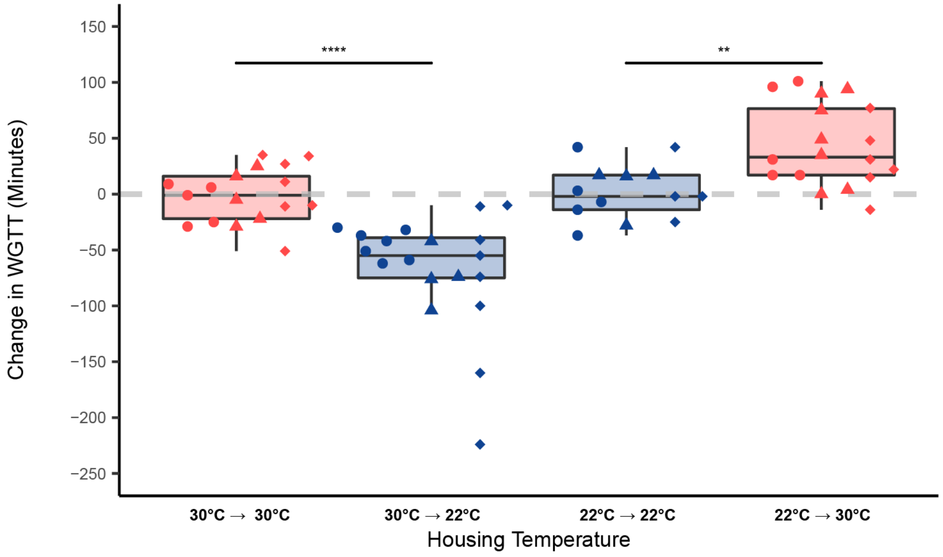

Figure S6. Gut transit time is altered by acclimation to a different housing temperature. The data from Fig. 3C and 3D combined into one panel. Difference in whole gut transit time before and after an acclimation period of two weeks to the same temperature, or a shift to a different temperature, for individual C57BL/6 mice determined by carmine red dye assay. Each point represents a measurement from an individual mouse. Shapes (●, ◆, ▲) indicate the independent experiment in which a measurement was taken. Boxes indicate the upper and lower quartiles, midline represents the median, and whiskers indicate non-outlier minima and maxima. Significance test results indicated by * $p < 0.05$, ** $p < 0.01$, *** $p < 0.001$, **** $p < 0.0001$. Extended statistics can be found in Supplementary Data 2. From left to right: change in whole gut transit time observed in 30°C mice after a two-week acclimation period either remaining at 30°C (red) or being transferred to 22°C (blue) as determined by the difference in carmine red passage time ($N_{30°C \rightarrow 30°C}$ = 17 (7 F, 10 M), $N_{30°C \rightarrow 22°C}$ = 19 (5 F, 14 M),; Wilcoxon test, W = 304.5, p = 6.27E-6); change in whole gut transit time observed in 22°C mice after a two-week acclimation period either remaining at 22°C (blue) or being transferred to 30°C (red) as determined by the difference in carmine red passage time ($N_{22°C \rightarrow 22°C}$ = 13 (8 F, 5 M), $N_{22°C \rightarrow 30°C}$ = 18 (9 F, 9 M); T-test, t = -3.82, df = 28.9, p = 6.59E-4).
