# Supplementary material for: Temperature-dependent differences in mouse gut motility are mediated by stress
Source: Lab Anim (NY). 2024 May 28;53(6):148–59. doi: 10.1038/s41684-024-01376-5 (PMC11147774; doi:10.1038/s41684-024-01376-5)
Supplement: Supplementary file 2 — Reporting Summary [file 41684_2024_1376_MOESM2_ESM.pdf]

nature portfolio

Corresponding author(s): Denise Monack, Ami Bhatt

Last updated by author(s): 01/13/2024

Reporting Summary

Nature Portfolio wishes to improve the reproducibility of the work that we publish. This form provides structure for consistency and transparency in reporting. For further information on Nature Portfolio policies, see our Editorial Policies and the Editorial Policy Checklist.

Statistics

For all statistical analyses, confirm that the following items are present in the figure legend, table legend, main text, or Methods section.

n/a	Confirmed	
☐	☒	The exact sample size (*n*) for each experimental group/condition, given as a discrete number and unit of measurement
☐	☒	A statement on whether measurements were taken from distinct samples or whether the same sample was measured repeatedly
☐	☒	The statistical test(s) used AND whether they are one- or two-sided<br>*Only common tests should be described solely by name; describe more complex techniques in the Methods section.*
☒	☐	A description of all covariates tested
☐	☒	A description of any assumptions or corrections, such as tests of normality and adjustment for multiple comparisons
☐	☒	A full description of the statistical parameters including central tendency (e.g. means) or other basic estimates (e.g. regression coefficient) AND variation (e.g. standard deviation) or associated estimates of uncertainty (e.g. confidence intervals)
☐	☒	For null hypothesis testing, the test statistic (e.g. F, t, r) with confidence intervals, effect sizes, degrees of freedom and P value noted<br>*Give P values as exact values whenever suitable.*
☒	☐	For Bayesian analysis, information on the choice of priors and Markov chain Monte Carlo settings
☒	☐	For hierarchical and complex designs, identification of the appropriate level for tests and full reporting of outcomes
☒	☐	Estimates of effect sizes (e.g. Cohen's *d*, Pearson's *r*), indicating how they were calculated

Our web collection on statistics for biologists contains articles on many of the points above.

Software and code

Policy information about availability of computer code

Data collection	No software was used for data collection, except Zen 2009 software for confocal microscopy.
Data analysis	R (4.2.1) was used for statistical analyses of all data. CellProfiler was used to quantify fluorescence intensity in the in situ staining experiment. Publicly available code used to classify sequencing data and perform downstream analyses are available at https://github.com/bhattlab/kraken2_classification/tree/master.

For manuscripts utilizing custom algorithms or software that are central to the research but not yet described in published literature, software must be made available to editors and reviewers. We strongly encourage code deposition in a community repository (e.g. GitHub). See the Nature Portfolio guidelines for submitting code & software for further information.

Data

Policy information about availability of data

All manuscripts must include a data availability statement. This statement should provide the following information, where applicable:

- Accession codes, unique identifiers, or web links for publicly available datasets
- A description of any restrictions on data availability
- For clinical datasets or third party data, please ensure that the statement adheres to our policy

Source data is available in Han_et_al_SourceData.xlsx. Sequencing data is available under SRA BioProject accession PRJNA1069241. The microscopy files from Fig. 5d and videos from Fig. 4 have been deposited on the Stanford Digital Repository at https://doi.org/10.25740/sc811vz1270.

Human research participants

Policy information about studies involving human research participants and Sex and Gender in Research.

Reporting on sex and gender	*Use the terms sex (biological attribute) and gender (shaped by social and cultural circumstances) carefully in order to avoid confusing both terms. Indicate if findings apply to only one sex or gender; describe whether sex and gender were considered in study design whether sex and/or gender was determined based on self-reporting or assigned and methods used. Provide in the source data disaggregated sex and gender data where this information has been collected, and consent has been obtained for sharing of individual-level data; provide overall numbers in this Reporting Summary. Please state if this information has not been collected. Report sex- and gender-based analyses where performed, justify reasons for lack of sex- and gender-based analysis.*
Population characteristics	*Describe the covariate-relevant population characteristics of the human research participants (e.g. age, genotypic information, past and current diagnosis and treatment categories). If you filled out the behavioural & social sciences study design questions and have nothing to add here, write "See above."*
Recruitment	*Describe how participants were recruited. Outline any potential self-selection bias or other biases that may be present and how these are likely to impact results.*
Ethics oversight	*Identify the organization(s) that approved the study protocol.*

Note that full information on the approval of the study protocol must also be provided in the manuscript.

Field-specific reporting

Please select the one below that is the best fit for your research. If you are not sure, read the appropriate sections before making your selection.

☒ Life sciences ☐ Behavioural & social sciences ☐ Ecological, evolutionary & environmental sciences

For a reference copy of the document with all sections, see nature.com/documents/nr-reporting-summary-flat.pdf

Life sciences study design

All studies must disclose on these points even when the disclosure is negative.

Sample size	No sample size calculations were performed; sample sizes used are at least 5 animals per group where possible, which is a common rule of thumb for animal experiments. Where possible, more animals were used to improve robustness of findings.
Data exclusions	Mice were excluded from data if they displayed signs of obvious distress or injury, or otherwise met criteria for early euthanasia. Samples were also excluded if they not meet kit requirements (e.g. if blood was hemolysed, ELISA kits recommended against using serum). Exclusion criteria were pre-established.
Replication	In vivo experiments were performed three times using different cohorts of mice to illustrate replicability where possible. Plots separate points by independent experiments where this was performed.
Randomization	Breeder animals used to establish the colony were randomly divided. For animals in temperature-swap experiments and antibiotic-treatment experiments, control and experimental groups were selected on a cage by cage basis. In astressin experiments, animals were stratified such that roughly 50% of animals were in control and 50% of animals were in treatment groups, and so that each cage had a control and treated animal.
Blinding	Blinding was not possible, as the first author performed most experiments alone and handling the mice necessitated knowing what groups they belonged to. Where possible, during image acquisition and analysis, and video analysis, authors were blinded to treatment groups.

Reporting for specific materials, systems and methods

We require information from authors about some types of materials, experimental systems and methods used in many studies. Here, indicate whether each material, system or method listed is relevant to your study. If you are not sure if a list item applies to your research, read the appropriate section before selecting a response.

Materials & experimental systems

n/a	Involved in the study
☒	☐ Antibodies
☒	☐ Eukaryotic cell lines
☒	☐ Palaeontology and archaeology
☐	☒ Animals and other organisms
☒	☐ Clinical data
☒	☐ Dual use research of concern

Methods

n/a	Involved in the study
☒	☐ ChIP-seq
☒	☐ Flow cytometry
☒	☐ MRI-based neuroimaging

Animals and other research organisms

Policy information about studies involving animals; ARRIVE guidelines recommended for reporting animal research, and Sex and Gender in Research

Laboratory animals	Breeder pairs of C57BL/6NJ (RRID:IMSR_JAX:005304) and 129x1/SvJ (RRID:IMSR_JAX:000691) were obtained from The Jackson Laboratories. Breeder pairs of C57BL/6J-Crh+/- mice were generously donated by Dr. Joseph Majzoub at Boston Children's Hospital (Zhang et al., 2017)
Wild animals	No wild animals were used.
Reporting on sex	Sex of each animal is included in source data in Han_et_al_SourceData.xlsx.
Field-collected samples	No field samples were collected.
Ethics oversight	Stanford APLAC Protocol 12826 approved experiments performed for this study.

Note that full information on the approval of the study protocol must also be provided in the manuscript.
